# Relapsed Chronic Lymphocytic Leukaemia with Concomitant Extensive Chronic Graft versus Host Disease after Allogeneic Haematopoietic Stem Cell Transplantation Successfully Treated with Oral Venetoclax

**DOI:** 10.1155/2021/8831125

**Published:** 2021-01-22

**Authors:** Ching Soon Teoh, Ai Sim Goh

**Affiliations:** Haematology Unit, Department of Internal Medicine, Hospital Pulau Pinang, Jalan Residensi, 10990 Georgetown, Penang, Malaysia

## Abstract

A middle-aged gentleman who was diagnosed with high-risk chronic lymphocytic leukaemia (CLL), Rai stage IV, Binet C with del(17p) and del(13q) underwent allogeneic haematopoeitic stem cell transplantation (allo-HSCT) from a human leukocyte antigen (HLA) identical sister. The patient developed extensive skin, oral, and liver chronic graft versus host disease (GVHD) required tacrolimus, mycophenolate mofetil (MMF), and prednisolone. At seventh month after allo-HSCT, the patient presented with systemic symptoms, right cervical lymphadenopathy, splenomegaly, marked pancytopaenia, and elevated lactate dehydrogenase (LDH). Bone marrow study, immunophenotyping (IP), chromosome analysis, and PET-CT scan confirmed relapsed CLL with no evidence of Richter's transformation or posttransplant lymphoproliferative disease (PTLD). Withdrawal of immunosuppressant (IS) worsened cutaneous and liver GVHD. Chemotherapy was not a suitable treatment option in view of immunodeficiency. The patient underwent extracorporeal photopheresis (ECP) therapy eventually for extensive chronic GVHD, and the IS were gradually tapered to the minimal effective dose. The relapsed CLL was treated successfully with oral venetoclax accessible via a compassionate drug program. This case highlights challenges in managing relapsed CLL and loss of graft-versus-leukaemia (GVL) effect despite extensive chronic GVHD. Venetoclax is an effective and well-tolerated oral novel agent for relapsed CLL after allo-HSCT.

## 1. Introduction

Chronic lymphocytic leukaemia (CLL) is the most common leukaemia in adults, and it remains an incurable malignancy with conventional chemoimmunotherapy [1]. Approximately 80% of CLL patients have cytogenetic abnormalities detected by fluorescence in situ hybridization (FISH) [2]. Detection of high-risk cytogenetic is important because it is associated with advanced disease, chemotherapy resistance, and inferior survival. To date, there are three new orally available pathway inhibitors [3] approved for relapsed or refractory CLL: the Bruton's kinase inhibitor, ibrutinib [4], the phosphatidylinositol 3-kinase (PI3K) inhibitor, idelalisib [5], and the B-cell lymphoma 2 (BCL-2) inhibitor, venetoclax [6, 7]. However, it is costly and not widely available in public hospitals. Allogeneic haematopoeitic stem cell transplantation (allo-HSCT) remains the only treatment with curative potential but is associated with high nonrelapsed mortality (NRM) [8, 9]. Therefore, only selected patients with high-risk disease should be considered for allografting. These poor prognostic markers include del(17p), *TP53* mutation, del(11q), complex cytogenetic abnormalities, and unmutated IgVH genes [10–12]. Remarkable improvement in patient outcomes has been achieved through reduced intensity conditioning (RIC) allo-HSCT for patients with CLL. There were less toxicity and NRM compared to myeloablative conditioning (MAC) [13–16]. CLL is a malignancy with a well-established responsiveness to graft-versus-leukaemia (GVL) effects, and GVL activity has been reported in several studies using RIC regimens. Complete response and disease clearance were successfully demonstrated following donor lymphocyte infusion (DLI) given for persistent mixed chimerism [17, 18]. Besides, the onset of chronic graft versus host disease (GVHD) following RIC allo-SCT was significantly correlated with conversion from minimal residual disease (MRD) detectable to undetectable status. Although overall outcomes have improved significantly with the use of RIC allo-HSCT for high-risk CLL patients, 35-50% of patients will still experience disease relapse [15, 16]. Posttransplant relapse may be successfully managed by immunosuppressant (IS) withdrawal, DLI, combined chemotherapy, and DLI or clinical trials involving novel agents. Data on patients with relapsed disease after allo-HSCT are limited, as most clinical trials have excluded this subset of patients from enrolment. In this case report, we describe a challenging case of relapsed CLL despite extensive chronic GVHD successfully treated with oral venetoclax. There was a significant improvement in skin, oral, and liver GVHD after extracorporeal photopheresis (ECP) therapy. Some of the IS were tapered successfully to a minimal effective dose without exacerbation of GVHD.

## 2. Case Report

A 45-year-old gentleman with no known medical illness presented to hospital with a two-week history of fever, symptomatic anaemia, abdominal distension, and multiple lymphadenopathies. Complete blood count showed leukocytosis of 43.7 × 10^9^/L, haemoglobin 2.5 g/dL, and platelet 59 × 10^9^/L. Lactate dehydrogenase (LDH) was 747 U/L (range < 250 U/L), and Coombs' test was negative. The haptoglobin test was not available in the hospital. Peripheral blood film revealed 96% small mature lymphocytes, 4% prolymphocytes, and numerous smudge cells. Bone marrow smears reported hypercellular marrow with lymphocytosis. The lymphocytes were small to medium in size with clumped chromatin and inconspicuous nucleoli. The immunophenotyping (IP) showed 58% of lymphocytes expressed CD19+, CD5+, CD23+, CD25+, FMC7–, and lambda restriction. There were 17p13.1 and 13q14.2 deletions from FISH analysis. Contrast-enhanced computed tomography (CECT) of the neck, thorax, abdomen, and pelvis showed extensive lymphadenopathies in bilateral cervical, supraclavicular, paratracheal, paraaortic, and inguinal with mild splenomegaly (13.6 cm). The biggest lymph node was found at the right cervical, measured 35 × 16 × 69 mm. The diagnosis of CLL Rai IV, Binet C with del(17p) and del(13q) was established.

The patient received ibrutinib covered by medical insurance for 4 months and achieved normal full blood count and LDH. There were no smudge cells or abnormal lymphocytes in the blood smear. Repeat CECT of the neck, thorax, abdomen, and pelvis showed complete regression of lymphadenopathies and splenomegaly. However, IP on the repeat bone marrow revealed residual 14% abnormal lymphocytes. The disease was in partial remission according to the International Workshop on CLL (iwCLL) 2018 guidelines. Owing to high-risk CLL with 17p deletion, a peripheral blood allo-HSCT from an HLA-identical sibling was recommended. The RIC regimen consisted of fludarabine 30 mg/m^2^ for 5 days, intravenous busulfan 3.2 mg/kg/day for 2 days, and cyclophosphamide 60 mg/kg/day for 1 day. The donor's peripheral blood stem cell (PBSC) was mobilised by granulocyte colony-stimulating factor (G-CSF) at the dose of 10 mcg/kg/day for 3 days. A total of 5.03 × 10^6^ CD34 per recipient body weight was infused. GVHD prophylaxis consisted of cyclosporine (CSA) and mycophenolate mofetil (MMF). The patient achieved neutrophil and platelet engraftment on day 15 after allo-HSCT.

On day 30 after allo-HSCT, the patient experienced steroid-refractory grade III acute GVHD of the skin and liver, which were treated with methylprednisolone, tacrolimus, ruxolitinib, and MMF. Cytomegalovirus polymerase chain reaction (CMV PCR) was detected on day 31 (937 copies/mL), with a peak level on day 40, measuring 57190 copies/mL. Intravenous immunoglobulin (IV IG) and oral valganciclovir were started since day 31 for CMV infection. It was difficult to differentiate liver GVHD from CMV hepatitis as liver biopsy was not performed due to thrombocytopenia. However, liver function improved while on IS, IV IG, and antiviral. Antihepatitis C virus, hepatitis B surface antigen, and total core antibody were not detected. Ultrasound of the hepatobiliary system showed no gallbladder obstruction or focal lesions in the liver. Skin biopsy confirmed cutaneous GVHD. He was discharged home after two months of stay in the ward. CMV infection resolved while ruxolitinib was discontinued during outpatient follow-up. Bone marrow smears, IP, trephine biopsy, and PET-CT scan on day 100 revealed disease in complete remission. Nevertheless, there was no repeat cytogenetic or FISH study due to an insufficient sample. There was difficulty in tapering tacrolimus, steroid, and MMF due to persistent lichenoid lesions at the buccal mucosa, hyperkeratotic plaques at the soft palate, recurrent oral ulcers, limited oral aperture due to sclerosis, poikiloderma, palms and feet desquamation, lichen planus like skin eruption, and persistent liver transaminitis. The device for ECP was not available at that time.

**Figure 1:**
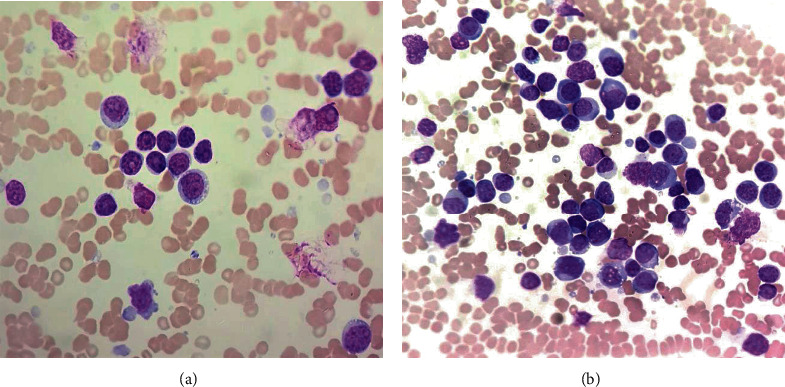
(a) Blood smear showed small lymphocytes, smudged and large lymphoid cells. (b) Large immature lymphoid cells with prominent nucleoli and basophilic vacuolated cytoplasm in the bone marrow smear.

Unfortunately, at the seventh month after allo-HSCT, he was readmitted to hospital with fever, poor appetite, right cervical lymphadenopathy, mild splenomegaly, pancytopenia, and markedly raised LDH. Complete blood count showed leukocytes 3.3 × 10^9^/L, haemoglobin 9.1 g/dL, and platelet 16 × 10^9^/L. The LDH was 1750 U/L (range < 250 U/L), and Coombs' test was negative. Bone marrow smears revealed 57% small lymphocytes, 23% abnormal lymphoid cells which were moderate to large with round oval nuclei, clumped chromatin, and scanty to moderate bluish cytoplasm (Figure 1). Some of the large abnormal lymphoid cells exhibited minimal cytoplasmic vacuolation. Both small lymphocytes and large lymphoid cells showed similar immunophenotypic expression by flow cytometry. The abnormal lymphocyte population expressed CD19+, CD5+, CD23+, CD25+, and lambda restriction. There is negative expression for CD20, CD10, CD22, smIgM, FMC7, CD38, CD108, CD11c, T, and N/K markers. EBER stain on trephine biopsy was negative. Cytogenetic analysis reported many numerical and structural abnormalities with 40-44, single X-chromosome in 12 cells analysed with no 17p deletion detected. FISH analysis was not performed. PET-CT scan showed FDG-avid uptake at bilateral cervical, right supraclavicular, left inguinal lymph nodes, and diffuse uptake in the spleen and bone marrow. No lymph node biopsy was performed due to severe thrombocytopenia. MMF was withdrawn. Oral tacrolimus and prednisolone were tapered gradually, but the patient experienced severe recurrent oral ulcers, sclerosis, and skin desquamation. Owing to worsening of chronic GVHD, the patient underwent ECP eventually at a weekly basis. He also received venetoclax 400 mg once daily following dose ramp-up with no evidence of tumour lysis syndrome. Systemic symptoms and peripheral lymphadenopathies resolved within four weeks of venetoclax. The blood count and LDH normalised. No abnormal lymphoid and smudge cells were seen in the blood film. Cutaneous and liver GVHD improved after the fifth cycle of ECP, and the IS were tapered successfully to the minimal effective dose. We plan to repeat bone marrow examination and PET-CT scan after six months of venetoclax.

## 3. Discussion

This is a young adult who is transplanted eligible for high-risk CLL. Many studies showed that the presence of del(17p) in CLL confers chemoimmunotherapy resistance and inferior survival [19, 20]. Fludarabine combined with cyclophosphamide and rituximab (FCR) remains the standard of practice for CLL in the developing countries given the drugs are readily available and affordable. However, FCR has failed to show significant efficacy among CLL patients with del(17p). The emergence of novel agents recently has changed the treatment paradigm for high-risk CLL [15, 21, 22]. The choice to proceed with allo-SCT or continue novel agents is a matter of debate nowadays. In our case, the patient received ibrutinib as the upfront therapy for CLL. It is generally a well-tolerated oral drug with rapid and durable responses without major adverse effects such as bleeding, atrial fibrillation, recurrent upper respiratory tract infection, and diarrhoea. Counts recovered and lymphadenopathies resolved after two months of therapy. In view of insufficient medical insurance coverage, the patient could only afford ibrutinib for four months before proceeding with RIC allo-HSCT.

Allo-HSCT is the only therapy with curative potential in CLL. The European Society for Bone and Marrow Transplantation (EBMT) recommends allo-HSCT for high-risk CLL with nonresponse or early relapse after purine analogues, relapse within 24 months after purine analogue combination therapy or treatment of similar efficacy and failure on at least one pathway inhibitor [23]. Our patient achieved complete remission after allografting but with considerable transplant-related toxicity. The patient developed steroid-refractory grade III acute GVHD required multiple IS followed by extensive chronic GVHD after 100 days post-allo-SCT. There is substantial evidence that GVL is effective in CLL, which include long-term molecular remission that can be achieved with allo-HSCT but not with autologous SCT [24], low risk of relapse in the presence of chronic GVHD [13, 17], an increased relapse risk with T-cell-depleted allografts [18], and efficacy of DLI [18, 25]. Although the crucial therapeutic principle of allo-SCT in CLL is GVL activity, the disease relapses eventually in this case. Loss of GVL activity is probably due to clonal evolution occurred at GVL sanctuary sites, which are not accessible to the GVL effect [26]. In addition, there was a loss of the Y-chromosome in the relapsed clone after sex-mismatched allo-HSCT. Minor histocompatibility antigens on the Y-chromosome have been identified as important targets for the GVL [27, 28]. Loss of Y-chromosome probably contributes to loss of GVL effect in our patient.

**Figure 2:**
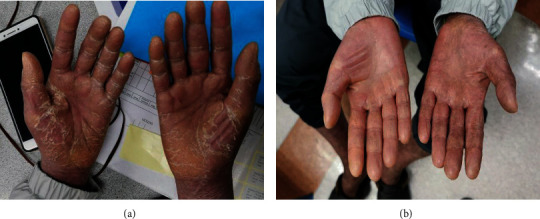
(a) Erythema and desquamation seen over both palms due to cutaneous chronic GVHD. (b) Significant improvement in cutaneous GVHD after ECP therapy.

IS withdrawal with or without DLI is commonly considered in relapsed CLL after allo-SCT, but this approach is not feasible in our patient with extensive chronic GVHD. To overcome issues related to IS withdrawal, a decision for ECP therapy was made. The procedure of ECP involves ultraviolet A (UVA) irradiation of autologous peripheral blood mononuclear cells (PBMC) which have been collected by leukapheresis and exposed to the photosensitizing drug called 8-methoxypsoralen (8-MOP) [29]. The photoactive PBMC is subsequently reinfused into the patient, thus reduce donor T-cell-mediated immune responses in GVHD. In our case, the patient received ECP therapy at a weekly basis and there was a significant improvement in chronic GVHD after fifth ECP. There was no longer grade III liver transaminitis, oral sclerosis, palm and feet desquamation, and lichen planus like skin eruption (Figure 2). The tacrolimus was kept at 1 mg twice a day after the seventh cycle of ECP therapy, and oral prednisolone was reduced to 15 mg daily. ECP therapy is extremely valuable in this case as it enables IS to be tapered to the lowest effective dose for chronic GVHD in the circumstances of relapsed CLL. Besides, it is a safe procedure and complications such as vasovagal attack and catheter-related blood stream infection are infrequent.

Our patient was commenced on venetoclax through a compassionate use program. Venetoclax is a highly selective inhibitor of BCL-2, an antiapoptotic protein that is overexpressed and allows CLL cells to evade apoptosis by sequestering proapoptotic proteins. Venetoclax is indicated as monotherapy for patients with CLL with or without del(17p) and who have received at least one prior therapy [6, 7, 30]. In the MURANO trial, venetoclax plus rituximab significantly reduced the risk of progression or death by 83% compared with bendamustine plus rituximab [30, 31]. In subgroup analysis, patients who received venetoclax plus rituximab achieved deep responses as shown by MRD negativity rates in both the peripheral blood and bone marrow. Eradication of MRD is a significant predictor for both progression-free survival (PFS) and overall survival (OS) [30]. Recent updates reported after 48 months of follow-up, PFS benefit was sustained with fixed treatment duration (24 months) in venetoclax plus rituximab arm compared with bendamustine plus rituximab arm (6 cycles) [30]. Our patient experienced grade III neutropenia and pneumonia during the initial 5-week dose-titration phase, which resolved after granulocyte colony-stimulating factor (GCSF) and antibiotics. He also received a course of rituximab at fifth week of venetoclax, but it was discontinued due to recurrent pneumonia. Sputum culture grew *Klebsiella pneumoniae*. Sputum for acid-fast bacilli (AFB) direct smears was negative, and the culture for Mycobacterium tuberculosis was no growth. A high-resolution CT (HRCT) scan of the thorax excluded lung GVHD. There was neither tumour lysis syndrome nor exacerbation of chronic GVHD after initiation of venetoclax. Ibrutinib is another novel agent which is effective for relapsed CLL with chronic GVHD. However, it is costly, and the patient could not afford it.

In conclusion, we achieved an excellent outcome when treating a patient with relapsed CLL after allo-HSCT using oral venetoclax and concomitant ECP therapy for extensive chronic GVHD. The quality of life has improved significantly with current therapy. Venetoclax is a well-tolerated oral novel agent and long-term follow-up is important to ensure the disease achieves a sustained complete remission.

## Data Availability

No data were used to support this study. The figures used to support the findings of this study are included within the article.
